# Clinical Efficiency of Non-invasive Prenatal Screening for Common Trisomies in Low-Risk and Twin Pregnancies

**DOI:** 10.3389/fgene.2021.661884

**Published:** 2021-05-10

**Authors:** Yanfei Xu, Pengzhen Jin, Yu Lei, Yeqing Qian, Yuqing Xu, Miaomiao Wang, Jinglei Jin, Yixuan Yin, Minyue Dong

**Affiliations:** ^1^Department of Reproductive Genetics, Women’s Hospital, School of Medicine, Zhejiang University, Hangzhou, China; ^2^Department of endocrinology, People’s Hospital of Zhejiang Province, Hangzhou, China; ^3^Key Laboratory of Reproductive Genetics, Ministry of Education (Zhejiang University), Hangzhou, China; ^4^Prenatal Diagnosis Center, Hangzhou Women’s Hospital, Hangzhou, China; ^5^Key Laboratory of Women’s Reproductive Health of Zhejiang Province, Hangzhou, China

**Keywords:** non-invasive prenatal screening, twin pregnancy, low-risk pregnancy, trisomy, clinical efficiency

## Abstract

To evaluate the clinical efficiency of non-invasive prenatal screening (NIPS) for fetal aneuploidies in low-risk and twin pregnancies, patients who received NIPS in a tertiary university hospital were enrolled, and their clinical data, NIPS results and pregnancy outcomes were collected. Patients were divided into singleton and twin pregnancies, and then those with singleton pregnancies were divided into low- and high-risk pregnancies. Sensitivity, specificity, positive predictive value (PPV) and negative predictive value (NPV) were estimated. Comparisons were made on the clinical efficiency of NIPS between singleton and twin pregnancies, as well as between low- and high-risk pregnancies. Of 66,172 patients enrolled, 59,962 were eligible for analysis. The sensitivity, specificity and NPV were ≥ 99% in singleton and twin pregnancies. The PPVs were 90.4, 56.6, and 13.0% in singleton pregnancies, while 100, 33.3, and 0% in twin pregnancies for trisomy 21 (T21), trisomy 18 (T18) and trisomy 13 (T13), respectively (*P* > 0.05 for all). The PPVs were 97.4 and 90.0% in high-risk pregnancies, while 78.6 and 16.7% in low-risk pregnancies for T21 and T18, respectively (*P* < 0.05 for all). In summary, the performance of NIPS in singleton pregnancies was similar to that in twin pregnancies. NIPS can be recommended for all pregnancies regardless of the risks.

## Introduction

In 1997, Lo et al. found the presence of fetal DNA in maternal plasma (Lo et al., 1997). Then cell-free DNA-based non-invasive prenatal screening (NIPS) has been established to detect fetal aneuploidies (Chiu et al., 2011; Ehrich et al., 2011; Palomaki et al., 2011, 2012; Bianchi et al., 2012; Norton et al., 2012). This technology has been widely used and benefited a lot of women (Chandrasekharan et al., 2014). Previous studies have shown that NIPS has high sensitivity and specificity in detecting common fetal trisomies (trisomies 21, 18, and 13) (Ehrich et al., 2011; Bianchi et al., 2012; Porreco et al., 2014; Gil et al., 2017). A recent meta-analysis estimated that the sensitivity of NIPS was more than 99% for trisomy 21 (T21), and more than 97% for trisomy 18 (T18) and trisomy 13 (T13), with a high specificity of more than 99% for these trisomies, even for low-risk pregnancies (Rezaei et al., 2019). The positive predictive values (PPV) of NIPS was higher for T21 and T18 than for T13, but PPVs vary in different studies (Chiu et al., 2011; Bianchi et al., 2014; Norton et al., 2015; Petersen et al., 2017; van der Meij et al., 2019). Both the ACMG guideline in 2016 and the updated ACOG guideline in 2020 recommend NIPS as the most sensitive and specific screening for T21, T18 and T13 (Gregg et al., 2016; Rose et al., 2020).

However, most studies focused on the NIPS in singleton and high-risk pregnancies, such as pregnancies of advanced maternal age and high-risk standard screening (Chiu et al., 2011; Palomaki et al., 2011, 2012; Norton et al., 2012). The updated ACOG guideline recommends NIPS be offered to all pregnant women regardless of maternal age or risk. Besides, it suggests NIPS be performed in twin pregnancies, and its performance for T21 was encouraging (Rose et al., 2020). However, the performances of NIPS in low-risk and twin pregnancies were not well understood up to now because of the limited positive cases of aneuploidies, especially the limited positive cases of T18 and T13 in low-risk and twin pregnancies. Meanwhile, few studies have compared the low-risk with the high-risk pregnancies on the detective efficiency of NIPS, and a similar problem exists in singleton and twin pregnancies. Thus, the clinical efficacy research of NIPS in low-risk and twin pregnancies is of great significance for the effective application of NIPS at the present stage.

In the current investigation, 66,172 pregnant women were enrolled in a single-centered tertiary university hospital. The first objective was to evaluate the clinical efficiency of NIPS for fetal T21, T18, and T13 in the overall patients. The second objective was to compare the performance between singleton and twin pregnancies, as well as between high- and low-risk pregnancies. This study will contribute to a more comprehensive understanding of the clinical efficiency of NIPS and reduce unnecessary worries for expanding the scope of application.

## Materials and Methods

### Participants and Data Collection

A retrospective single-centered study was conducted in Women’s Hospital, Zhejiang University School of Medicine, and 66,172 pregnant women who received NIPS were enrolled from February 2, 2015, to December 31, 2019. This study was approved by the Ethics Committee of Women’s Hospital, Zhejiang University School of Medicine, and all the patients provided their informed consent.

Clinical counseling was provided for all the patients before NIPS was provided. Patients with low-risk NIPS were advised to continue the routine prenatal care, and those with high-risk were advised to receive genetic counseling and invasive prenatal diagnosis. The diagnosis of T21, T18, and T13 depends on the karyotyping and physical examination of the newborn. Pregnancy outcomes of all cases were followed up.

The clinical data included gestational age at sampling, maternal age at the expected date of delivery, fetal numbers, results of standard screening, ultrasonography, NIPS risks, karyotypes and pregnancy outcomes, etc. The standard screening included the first or second-trimester screening or the combined screening. The first-trimester screening included the measurement of serum biomarkers with or without nuchal translucency (NT). The abnormalities of ultrasound included: (1) micro-anomalies or soft markers, such as increased NT (≥2.5 mm), fetal choroid plexus cysts, echogenic intracardiac focus (EIF), nasal bone hypoplasia, nasal bone absence, polyhydramnios, oligohydramnios and single umbilical artery (SUA); (2) structural abnormalities, such as fetal hydrops fetalis, cystic hygroma and urinary tract anomalies. A full-time staff followed up pregnancy outcomes, and the information was obtained by questionnaires or phone calls after delivery.

### Study Design

In the primary analysis, sensitivity, specificity, PPV and negative predictive value (NPV) were estimated overall. Patients were divided into two groups: singleton and twin pregnancies, and NIPS performance was compared.

In the secondary analysis, singleton pregnancies were divided into three cohorts based on maternal age, results of standard screening and ultrasonography. In each cohort, patients were further divided into high- and low-risk pregnancies. Those with advanced maternal age, high risk of standard screening and abnormal ultrasonography were incorporated into the high-risk pregnancies, and those without the information above were the low-risk pregnancies. The performance was evaluated and comparisons were made.

### NIPS

The DNA extraction, library construction and sequencing were performed according to the protocol of the Human Molecular Genetics Guidelines (Jiang et al., 2012; Chen et al., 2013). Two hundred microliter maternal plasma was used for cell-free fetal DNA extraction by BGISP-300 (BGI, Shenzhen, China) and Nucleic Acid Extraction Kit (BGI, Shenzhen, China). End-repair was performed by adding end-repair enzymes, and PCR was used to amplify DNA. The DNA amplification products were quantified by Qubit^®^ 2.0 (Life Tech, Invitrogen, United States) using Qubit^TM^ dsDNA HS Assay Kits (Life Tech, Invitrogen, United States). The volume was calculated according to the concentration of each sample, and samples of the same mass were mixed by pooling. The DNA double strands were thermally denatured after pooling, and then the cyclic buffer and the ligase were added to make DNA circles by the cyclization reaction. DNA circles were used to make DNBs by rolling circle replication (RCR). The concentration of DNBs was quantified by Qubit 2.0 using Qubit^TM^ ssDNA Assay Kits (Life Tech, Invitrogen, United States) and the DNBs concentration in the range of 8–40 ng/μL was considered as appropriate concentration. The DNBs were loaded onto chips and sequenced on the BGISEQ-500 sequencing platform (BGI, Shenzhen, China). Any sample that failed to meet the quality control criteria (Supplementary Table 1) was reported as a detection failure.

The sequence from NGS was compared with the reference sequence map of the human genome, and the percentage of each chromosome was calculated by Illumina Sequencing Analysis Viewer1.9.1 software. The Z value was used to evaluate the actual disease situation of the samples.

### Karyotyping

Amniotic fluid or umbilical cord blood samples were collected. The karyotype was analyzed according to the standard of ISCN (2016) through the process of standardized cell culture, filmmaking and G-banding.

### Statistical Analysis

The SPSS statistical software package (version 25.0) was used for statistical analysis. For the analysis of sensitivity, specificity, PPV, NPV and the corresponding 95% confidence intervals (CI), Non-parametric Test of One Sample and the Clopper–Pearson method was used. Chi-square test of Crosstabulation, Pearson Chi-Square or Fisher’s Exact Test were used where appropriate. A *P*-value of less than 0.05 was considered to be of statistical significance.

## Results

### Study Participants

**FIGURE 1:**
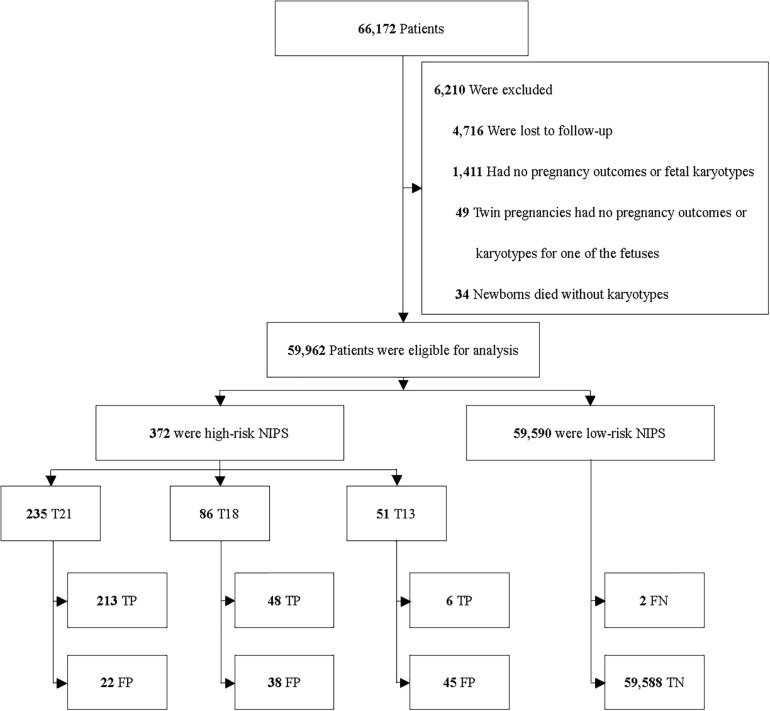
Enrollment and outcomes.

Among 66,172 enrolled patients, 4,716 (7.1%) were excluded because of the drop in follow-up. 1,494 (2.3%) were excluded due to the following situations: (1) missing the data of pregnancy outcomes; (2) missing the data of fetal karyotypes; (3) twin pregnancies but one fetus without the data of pregnancy outcomes or karyotypes; (4) newborns died without karyotypes or physical examinations. 59,962 patients were included for analysis (Figure 1).

**FIGURE 2:**
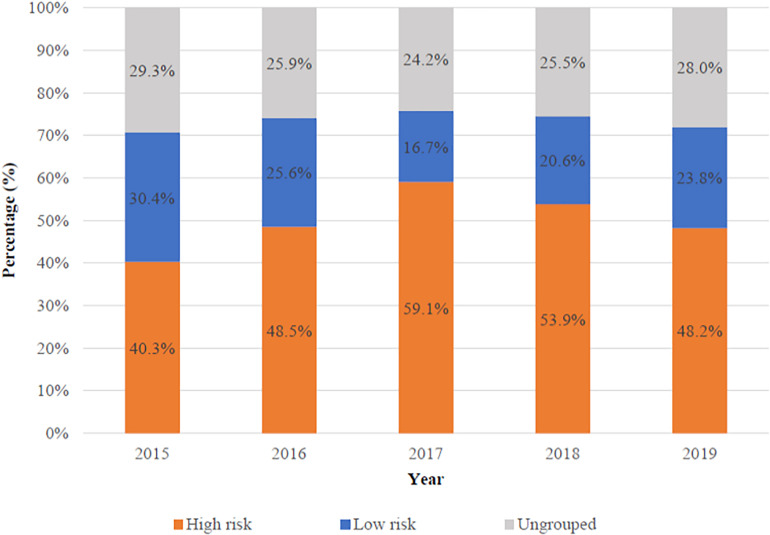
The proportion of high- and low-risk pregnancies varies with the year.

Most previous studies focused on the performance of NIPS in high-risk pregnancies. To respond to the updated ACOG guideline in 2020, we focused on low-risk pregnancies. The proportion of high- and low-risk pregnancies varies with the year was shown in Figure 2. High-risk pregnancies had one of the following factors: advanced maternal age, high risk of standard screening, abnormal ultrasonography and personal or family history of aneuploidy. Low-risk pregnancies had none of the high-risk factors above. There were 30,405 (50.7%) high-risk and 13,731 (22.9%) low-risk patients in all 59,962 patients, respectively. Besides, 15,826 (26.4%) patients could not be grouped because of missing the complete standard screening data, ultrasonography, or information of maternal age.

The clinical characteristics were presented in Table 1. The mean maternal age was 32 years old (range 15–60) at delivery, and the mean gestational age was 17^+^ weeks (range 12–37.4) at sampling. In all, 892 (1.5%) patients underwent karyotyping. Among them, 10 were postnatal testing. For the remaining patients, the pregnancy outcomes were based on the physical examinations of the newborns.

**TABLE 1:** Clinical characteristics.

Characteristics	Value
No.	59,962
Maternal age (range), years	32 (15−60)
Maternal age < 35 years, no. (%)	33,246 (55.4)
Maternal age ≥ 35 years, no. (%)	22,777 (38.0)
Gestational age (range), weeks	17^+^ (12−37.4)
First trimester (12–13.9 weeks), no. (%)	7,877 (13.1)
Second trimester (14–27.9 weeks), no. (%)	45,087 (75.2)
Third trimester (28–37.4weeks), no. (%)	410 (0.7)
Singleton, no. (%)^§^	57,563 (96.0)
Twin, no. (%)	2,399 (4.0)
IVF-ET, no. (%)	3,951 (6.6)
Spontaneous, no. (%)	49,389 (82.4)
Standard screening for T21 and T18, no. (%)	20,330 (33.9)
High risk	5,373 (9.0)
Low risk	14,957 (24.9)
Abnormal ultrasonography, no. (%)	2,741 (4.6)
Personal or family history of aneuploidy, no. (%)	148 (0.2)
Karyotype analysis, no. (%)	892 (1.5)

**^§^145 patients had vanished twin syndromes; IVF-ET, in vitro fertilization and embryo transfer.**

Among 59,962 patients, 269 (1 in 223) carried fetuses of aneuploidies that were confirmed by karyotyping. Among them, 215 were T21, 48 were T18 and six were T13, with a prevalence of 1 in 279 for T21, 1 in 1,249 for T18 and 1 in 9,994 for T13, respectively.

### Primary Analysis

#### Overall Performance

Among the 59,962 patients, 213 were true positive (TP), 22 were false positive (FP) and two were false negative (FN) for T21; 48 were TP, 38 were FP and none was FN for T18; six were TP, 45 were FP and none was FN for T13. The estimated sensitivity, specificity, PPV and NPV were listed in Table 2.

**TABLE 2:** Overall performance.

NIPS	TP	FP	TN	FN	Sensitivity	Specificity	PPV	NPV
			
	N				%, (95% CI)			
T21	213	22	59,725	2	99.1 (96.7–99.9)	100 (99.9–100)	90.6 (86.2–94.0)	100 (100–100)
T18	48	38	59,876	0	100 (92.6–100)	99.9 (99.9–100)	55.8 (44.7–66.5)	100 (100–100)
T13	6	45	59,911	0	100 (54.1–100)	99.9 (99.9–99.9)	11.8 (4.4–23.9)	100 (100–100)

**PPV, positive predictive value; NPV, negative predictive value.**

#### Performance in Singleton and Twin Pregnancies

Among the 57,563 women with singleton pregnancies, 206 were TP, 22 were FP and two were FN for T21; 47 were TP, 36 were FP and none was FN for T18; six were TP, 40 were FP and none was FN for T13. Among the 2,399 women with twin pregnancies, seven were TP, none was FP or FN for T21; one was TP, two were FP and none was FN for T18; none was TP, five were FP and none was FN for T13. The detective efficiency and comparisons between the two groups were listed in Table 3. Except for the specificity of T13 (*P* = 0.03), there was no statistical difference (*P* > 0.05 for all).

**TABLE 3:** NIPS performance in twin pregnancies.

NIPS	Singleton pregnancies	Twin pregnancies	*P*-value
No.	57,563	2,399	
**T21**			
TP (n)	206	7	
FP (n)	22	0	
TN (n)	57,333	2,392	
FN (n)	2	0	
Sensitivity,%, (95% CI)	99.0 (96.6–99.9)	100 (59.0–100)	1.00
Specificity,%, (95% CI)	100 (99.9–100)	100 (99.8–100)	1.00
PPV,%, (95% CI)	90.4 (85.8–93.9)	100 (59.0–100)	1.00
NPV,%, (95% CI)	100 (100–100)	100 (99.8–100)	1.00
**T18**			
TP (n)	47	1	
FP (n)	36	2	
TN (n)	57,480	2,396	
FN (n)	0	0	
Sensitivity,%, (95% CI)	100 (92.5–100)	100 (2.5–100)	*
Specificity,%, (95% CI)	99.9 (99.9–100)	99.9 (99.7–100)	0.66
PPV,%, (95% CI)	56.6 (45.3–67.5)	33.3 (0.8–90.6)	0.58
NPV,%, (95% CI)	100 (100–100)	100 (99.8–100)	*
**T13**			
TP (n)	6	0	
FP (n)	40	5	
TN (n)	57,517	2,394	
FN (n)	0	0	
Sensitivity,%, (95% CI)	100 (54.1–100)	Cannot be calculated	*
Specificity,%, (95% CI)	99.9 (99.9–100)	99.8 (99.5–99.9)	0.03
PPV,%, (95% CI)	13.0 (4.9–26.3)	0 (0–52.2)	1.00
NPV,%, (95% CI)	100 (100–100)	100 (99.8–100)	*

*The paired groups could not make comparisons.

### Secondary Analysis

About 96.0% (57,563 in 59,962) of women were singleton, and 97.0% (261 in 269) of T21, T18 and T13 were detected in singleton pregnancies. Of the three cohorts of maternal age, standard screening and ultrasonography in singleton pregnancies, the detective efficiency was estimated and comparisons were made on the clinical efficiency between low- and high-risk pregnancies (Table 4).

**TABLE 4:** NIPS performance in singleton pregnancies at different risks.

NIPS	Maternal age (years)		Standard screening^ϕ^		Ultrasonography		Low-risk pregnancies^ψ^
				
	< 35	≥ 35	Low-risk	High-risk	Normal	Abnormal	
No.	31,359	22,324	14,902	5,362	51,080	2,606	13,731
**T21**							
TP	101	96	22	38	151	46	19
FP	10	10	6	1	19	1	6
TN	31,247	22,217	14,873	5,323	50,910	2,559	13,706
FN	1	1	1	0	0	2	0
Sensitivity,%, (95% CI)	99.0 (94.7–100)	99.0 (94.4–100)	95.7 (78.1–99.9)	100 (90.7–100)	100 (97.6–100)	95.8 (85.7–99.5)	100 (82.4–100)
Specificity,%, (95% CI)	100 (99.9–100)	100 (99.9–100)	100 (99.9–100)	100 (99.9–100)	100 (99.9–100)	100 (99.8–100)	100 (99.9–100)
PPV,%, (95% CI)	91.0 (84.1–95.6)	90.6 (83.3–95.4)	78.6 (59.0–91.7)	97.4 (86.5–99.9)^¶^	88.8 (83.1–93.1)	97.9 (88.7–99.9)	76.0 (54.9–90.6)
NPV,%, (95% CI)	100 (100–100)	100 (100–100)	100 (100–100)	100 (99.9–100)	100 (100–100)	99.9 (99.7–100)^‡^	100 (100–100)
**T18**							
TP	22	23	3	9	35	11	3
FP	23	12	15	1	35	0	15
TN	31,314	22,289	14,884	5,352	51,010	2,595	13,713
FN	0	0	0	0	0	0	0
Sensitivity,%, (95% CI)	100 (84.6–100)	100 (85.2–100)*	100 (29.2–100)	100 (66.4–100)*	100 (90.0–100)	100 (71.5–100)*	100 (29.2–100)
Specificity,%, (95% CI)	99.9 (99.9–100)	99.9 (99.9–100)	99.9 (99.8–99.9)	100 (99.9–100)	99.9 (99.9–100)	100 (99.9–100)	99.9 (99.8–99.9)
PPV,%, (95% CI)	48.9 (33.7–64.2)	65.7 (47.8–80.9)	16.7 (3.6–41.4)	90.0 (55.5–99.7)^‡^	50.0 (37.8–62.2)	100 (71.5–100)^‡^	16.7 (3.6–41.4)
NPV,%, (95% CI)	100 (100–100)	100 (100–100)*	100 (100–100)	100 (99.9–100)*	100 (100–100)	100 (99.9–100)*	100 (100–100)
**T13**							
TP	3	2	0	1	3	2	0
FP	25	13	10	2	32	6	9
TN	31,331	22,309	14,892	5,359	51,045	2,598	13,722
FN	0	0	0	0	0	0	0
Sensitivity,%, (95% CI)	100 (29.2–100)	100 (15.8–100)*	Cannot be calculated	100 (2.5–100)*	100 (29.2–100)	100 (15.8–100)*	Cannot be calculated
Specificity,%, (95% CI)	99.9 (99.9–99.9)	99.9 (99.9–100)	99.9 (99.9–100)	100 (99.9–100)	99.9 (99.9–100)	99.8 (99.5–99.9)^‡^	99.9 (99.9–100)
PPV,%, (95% CI)	10.7 (2.3–28.2)	13.3 (1.7–40.5)	0 (0–30.8)	33.3 (0.8–90.6)	8.6 (1.8–23.1)	25.0 (3.2–65.1)	0 (0–33.6)
NPV,%, (95% CI)	100 (100–100)	100 (100–100)*	100 (100–100)	100 (99.9–100)*	100 (100–100)	100 (99.9–100)*	100 (100–100)

The comparisons were made between the paired groups on the sensitivity, specificity, PPV and NPV. The P-values (two-sided) were marked if there was a statistical significance; ^ϕ^the cut-off value was < 1/270 for T21 and < 1/350 for T18 in the low-risk group; the cut-off value was ≥ 1/270 for T21 and ≥ 1/350 for T18 in the high-risk group; ^ψ^patients had no high-risk factors, including maternal age, standard screening, ultrasonography and personal or family history of aneuploidy simultaneously; ^¶^ P < 0.05; ^‡^P < 0.01; *the paired groups could not make comparisons.

#### The Cohort of Maternal Age

Among the 31,359 patients who were under 35 years old at delivery (low-risk), 101 were TP, 10 were FP and one was FN for T21; 22 were TP, 23 were FP and none was FN for T18; three were TP, 25 were FP and none was FN for T13. Among the 22,324 patients who were 35 years old or older (high-risk), 96 were TP, ten were FP and one was FN for T21; 23 were TP, 12 were FP and none was FN for T18; two were TP, 13 were FP and none was FN for T13. There was no significant difference in detective efficiency between the two groups (*P* > 0.05 for all).

#### The Cohort of Standard Screening

Among the 14,902 patients who were at low risk of standard screening, 22 were TP, six were FP and one was FN for T21; three were TP, 15 were FP and none was FN for T18; none was TP, ten were FP and none was FN for T13. The incidence was 0.2% (23/14,902) for T21, 0.02% (3/14,902) for T18 and 0% (0/14,902) for T13, respectively. Among the 5,362 patients who were at high risk of standard screening, 38 were TP, one was FP and none was FN for T21; nine were TP, one was FP and none was FN for T18; one was TP, two were FP and none was FN for T13. The incidence was 0.7% (38/5,362) for T21, 0.2% (9/5,362) for T18 and 0.02% (1/5,362) for T13, respectively. The incidence of T21 and T18 was significantly higher in the high-risk than in the low-risk group (*P* < 0.01 for both). Except for the PPV of T21 (*P* = 0.02) and T18 (*P* ≤ 0.01), there was no statistical difference (*P* > 0.05) between the two groups.

#### The Cohort of Ultrasonography

Among the 51,080 patients who had normal ultrasonography (low-risk), 151 were TP, 19 were FP and none was FN for T21; 35 were TP, 35 were FP and none was FN for T18; three were TP, 32 were FP and none was FN for T13. The incidence was 0.3% (151/51,080) for T21, 0.1% (35/51,080) for T18 and 0.01% (3/51,080) for T13, respectively. Among the 2,606 patients who had abnormal ultrasonography (high-risk), 46 were TP, one was FP and two were FN for T21; 11 were TP, none was FP and none was FN for T18; two were TP, six were FP and none was FN for T13. The incidence was 1.8% (48/2,606) for T21, 0.4% (11/2,606) for T18 and 0.1% (2/2,606) for T13, respectively. The incidence of T21, T18 and T13 was higher in the high-risk than in the low-risk group (*P* < 0.01 for T21 and T18; *P* = 0.02 for T13). Except for the NPV of T21 (*P* ≤ 0.01), PPV of T18 (*P* ≤ 0.01) and specificity of T13 (*P* ≤ 0.01), there was no statistical difference (*P* > 0.05) between the two groups.

#### Performance in the Low-Risk Pregnancies

Among the 13,731 patients without any high-risk factors, 19 were TP, six were FP and none was FN for T21; 3 were TP, 15 were FP and none was FN for T18; none was TP, nine were FP, and none was FN for T13.

### False-Negative Cases

There were two false-negative cases of singleton pregnancies. For case 1, the woman was 29 years old with a body mass index (BMI) of 27.3 and a normal karyotype. She had a low-risk standard screening. NIPS was conducted at the gestational age of 13 weeks. The fetal fraction of DNA was unknown. For case 2, the woman was 39 years old with a BMI of 24.8 and a normal karyotype. NIPS was conducted at the gestational age of 13^+^ weeks. The fetal fraction of DNA was 7%. No other high-risk factors were found before the screening except for the increased NT (3 mm for case 1 and 7.1 mm for case 2). It was confirmed that the karyotype of case 1 was 47, XX, + 21(15)/46, XX(85). Case 2 underwent the prenatal diagnosis because of the abnormal ultrasonography of hydroncus and complete cardiac cushion defect, and the confirmed karyotype was 47, XN, +21.

## Discussion

In this retrospectively single-centered study, we found that NIPS had high sensitivity, specificity and NPV for detecting common fetal trisomies in pregnancies with all risk levels, which was similar to a multicenter prospective study in China (Zhang et al., 2015). The PPV of NIPS was highest for T21 (90.6%), moderate for T18 (55.8%), and relatively low for T13 (11.8%). Our study included singleton and twin pregnancies, as well as pregnancies of all risk levels. We found that the performance of NIPS in singleton pregnancies was similar to that in twin pregnancies. With the higher incidence of T21 and T18 in high-risk pregnancies, the PPV was higher in high-risk than in low-risk pregnancies, but the high sensitivity, specificity and NPV were shown in both high- and low-risk pregnancies. Two false-negative cases of T21 were identified in pregnancies with abnormal ultrasonography, indicating that the pregnancies with abnormal ultrasonography should receive the prenatal diagnosis. The confirmed mosaic T21 indicates that fetal mosaicism is an important cause of false-negative NIPS.

In the current study, the high-risk patients were the main population for NIPS, indicating that NIPS has mainly been applied for or chosen by high-risk pregnant women, and this is inconsistent with the 2020 ACOG guidelines. One reason for this might be that patients who were at high risk would like to choose the NIPS first. The second reason might be the marked increase in pregnant women with advanced maternal age due to the two-child policy in China (Tian et al., 2020). NIPS had a similar performance on sensitivity, specificity, PPV and NPV in different maternal ages in the current observation, and it was consistent with a previous report, but they only analyzed the sensitivity (Dar et al., 2014).

Generally, the incidence of fetal trisomies was higher in the high-risk than in the low-risk pregnancies (Nicolaides et al., 2012). In our study, the incidence of fetal T21 was higher in the high-risk than in the low-risk standard screening, and the same was found for T18 in standard screening and ultrasonography cohorts. The higher incidence might explain the higher PPV in high-risk pregnancies because the PPV is associated with the number of positives cases in the population (Neufeld-Kaiser et al., 2015; Wang et al., 2015). In our study, the PPVs for T21, T18, and T13 were lower than a multicenter prospective study in China (Zhang et al., 2015), and lower than a national study in the Netherlands in which the PPV was 96% for T21, 98% for T18 and 53% for T13, respectively (van der Meij et al., 2019). The possible reason is that the first study was performed begin 2012 when the enrolled cases were mainly high-risk pregnancies. Therefore, they had a higher incidence of aneuploidies (1/155, 1/663, and 1/5,121 for T21, T18, and T13, respectively) and higher PPVs. The second study had a higher follow-up rate than ours, especially for cases with a high-risk NIPS. While in our cohort, some cases that had a high-risk NIPS rejected karyotyping or were lost to follow-up, and these cases might carry fetal trisomies. Considering those, the incidence of trisomies in our cohort may be lower than it was, so as PPV. Even though the higher PPV in high-risk pregnancies, we could not directly conclude that NIPS performs better in high-risk pregnancies because the ability of the test is determined by the precision of the technology and fetal fractions in the maternal blood rather than the incidence of trisomies in the population (Ashoor et al., 2012; Nicolaides et al., 2012). Additionally, the high specificity and NPV in each group of the three cohorts as well as in low-risk pregnancies showed the excellent ability of NIPS in excluding the unaffected pregnancies at any risk levels.

Herein, we proved the high sensitivity of NIPS in singleton and twin pregnancies, which is similar to previous reports (Gil et al., 2015, 2017). Due to the similar performance of NIPS in twin and singleton pregnancies (Fosler et al., 2017; Niemchak et al., 2020), some researchers considered that it could be used as a first-line screening in twin pregnancies (Le Conte et al., 2018; Yu et al., 2019). However, on account of the limited cases of trisomies in twin pregnancies, especially T18 and T13, the performance of NIPS may be unrepresentative and it needs to be further evaluated (Gil et al., 2019).

Two cases of false-negative T21 were identified in pregnancies with abnormal ultrasonography, and it lowed the NPV in this population. These discordant results highlight the irreplaceable status of early ultrasound in prenatal screening strategies (Hui et al., 2017), and NIPS is not a diagnostic test with inevitable false-positive and false-negative results (Allyse and Wick, 2018). More importantly, for those with abnormal ultrasonography, prenatal diagnosis instead of NIPS is essential, which has been issued in a previous study (Beulen et al., 2017), and it has been reported that the rate of pathogenic chromosome abnormalities missed by NIPS in the population of abnormal ultrasonography was 8% (Benachi et al., 2015), which was higher than ours (4%), but we only estimated the common trisomies. Causes of false-negative NIPS include low fetal fraction, which is caused by maternal obesity, multiple gestations causing low fetal fraction per fetus, maternal medical condition or treatment affecting the quality of circulating DNA, certain fetal chromosomal aneuploidies and confined placental mosaicism (Bianchi and Chiu, 2018). In this study, fetal mosaicism was confirmed by the karyotyping, causing the false-negative NIPS of case 1, and a similar finding has been reported in another study (Grati et al., 2014). For case 2, the fetal fraction was 7%, and it was a singleton pregnancy with a BMI of 24.8 and a normal karyotype. Meanwhile, there was no maternal medical condition or treatment during that time. A similar finding has been reported in an obvious study in which placental samples of two false-negative cases of T18 were obtained and both showed low-level confined placental mosaicism of T18 (Zhang et al., 2015). So, confined placental mosaicism may be the cause of false-negative NIPS of case 2.

There are several strengths in this study. Firstly, we assessed the performance of NIPS for common trisomies in a large population with different risk levels. Secondly, we make comparisons of singleton and twin pregnancies and confirmed a similar performance. Thirdly, we make comparisons of low- and high-risk populations in singleton pregnancies, and in which, the analysis was based on a single cohort of maternal age, conventional Down screening, or ultrasonography instead of the combined risk factors (Zhang et al., 2015). This study is the first large-scale retrospective study to assess the performance of NIPS in twin and low-risk pregnancies since the publication of the updated ACOG guideline.

There are also limitations in our study. Firstly, more than 4,400 patients lack information on maternal age, and some did not receive standard and ultrasound screening. So, we could not assess the risk levels of aneuploidy for these patients accurately. Secondly, we did not enroll cases that had no-call NIPS results and cases that had intrauterine fetal demise or selective termination of pregnancy, but they may be high risk for aneuploidy (Norton et al., 2015). From this point of view, our results may be little different from reality.

## Conclusion

NIPS has excellent clinical efficiency for common fetal trisomies in the overall patients at different risks, and its performance in singleton pregnancies was similar to that in twin pregnancies. However, NIPS cannot replace the invasive prenatal diagnosis, and fetuses with abnormal ultrasonography should undergo prenatal diagnosis.

## Data Availability Statement

The datasets presented in this study can be found in online repositories. The names of the repository/repositories and accession number(s) can be found below: doi: 10.6084/m9.figshare.14407616.

## Ethics Statement

This study was approved by the Ethics Committee of Women’s Hospital, Zhejiang University School of Medicine, and all the patients provided their informed consent.

## Author Contributions

MD and YaX: conception and design. YaX: statistical analysis and manuscript writing. MD: supervising and manuscript revising. All authors collected the data and approved the final version of the manuscript.

## Conflict of Interest

The authors declare that the research was conducted in the absence of any commercial or financial relationships that could be construed as a potential conflict of interest.
