# Supplementary material for: Clinical Efficiency of Non-invasive Prenatal Screening for Common Trisomies in Low-Risk and Twin Pregnancies
Source: Front Genet. 2021 May 10;12:661884. doi: 10.3389/fgene.2021.661884 (PMC8143437; doi:10.3389/fgene.2021.661884)
Supplement: Supplementary file 1 [file Table_1.DOC]

**Supplementary Table 1 Quality Control of Samples**

| Quality control | Reference values |
| --- | --- |
| Effective data volume (M) | [3.5, +∞) |
| GC (%) | [38, 42] |
| Q20 (%) | [93, 100] |
| Raw data volume (M) | [5.2, +∞) |
| Comparison rate (%) | [70, 100] |
| Repetitive rate (%) | [0, 12] |
| Abnormal chromosome number | [0, 3] |
| Fetal fraction (%) | [3.5, 100] |
| Fail_Rate (%) | [0, 40] |
| Total output (M) | [350, + ∞) |
| Resolution ratio (%) | [75, 100] |
| Dim_Rate (%) | [0, 4.5] |

Q20，quality threshold of 20
